# Fibrotic Fortresses and Therapeutic Frontiers: Pancreatic Stellate Cells and the Extracellular Matrix in Pancreatic Cancer

**DOI:** 10.1002/cam4.70788

**Published:** 2025-05-28

**Authors:** Sila Sigirli, Didem Karakas

**Affiliations:** ^1^ Medical Biotechnology, Graduate School of Health Acibadem Mehmet Ali Aydinlar University Istanbul Turkiye

**Keywords:** clinical trials, pancreatic cancer, pancreatic stellate cells, stroma, tumor microenvironment

## Abstract

**Background:**

Pancreatic ductal adenocarcinoma (PDAC) is characterized by a unique tumor microenvironment (TME) that plays pivotal roles in cancer progression, angiogenesis, metastasis, and drug resistance. This complex and dynamic ecosystem comprises cancer cells, stromal cells, and extracellular matrix (ECM) components, which interact synergistically to drive cancer aggressiveness. Among the stromal cells, cancer‐associated fibroblasts (CAFs) and pancreatic stellate cells (PSCs), mainly accepted as a group of CAFs, are central players in shaping the desmoplastic, hypoxic, and immunosuppressive stroma of PDAC. PSCs, the most abundant stromal cells in PDAC, are resident pancreatic cells that undergo phenotypic changes upon activation, driving tumor progression through the secretion of cytokines, growth factors, ECM components (e.g., collagen, hyaluronic acid, fibronectin), and matrix metalloproteinases. In addition to cellular elements, ECM components significantly contribute to cancer aggressiveness by forming a physical barrier that hinders drug penetration, activating signaling pathways through specific receptor interactions, and generating peptides originating from the fragmentation of proteins to induce cancer migration. Regarding their critical roles in tumor progression, therapeutic approaches targeting PSCs and the ECM have garnered increasing interest in recent years. However, PSCs and stromal components may exhibit dual roles, with the potential to both promote and suppress tumor progression under different conditions. Therefore, targeting PSCs or stroma may lead to unintended outcomes, including exacerbation of cancer aggressiveness.

**Methods:**

This review focuses on the multifaceted roles of PSCs in PDAC, particularly their interactions with cancer cells and their contributions to therapy resistance. Additionally, we discuss current and emerging therapeutic strategies targeting PSCs and the ECM components, including both preclinical and clinical efforts.

**Conclusion:**

By synthesizing insights from recent literature, this review provides a comprehensive understanding of the role of PSCs in PDAC pathobiology and highlights potential therapeutic approaches targeting PSCs or ECM components to improve patient outcomes.

## 1 Introduction

The unique tumor microenvironment (TME) is a characteristic feature of pancreatic ductal adenocarcinoma (PDAC) and plays a pivotal role in cancer progression, angiogenesis, metastasis, and drug resistance [1]. This highly complex and dynamic ecosystem comprises not only cancer cells but also various stromal cells, including fibroblasts, adipocytes, immune cells, and vascular cells, as well as extracellular components. These cellular and non‐cellular components of tumors interact extensively with each other and with cancer cells, collectively driving tumor aggressiveness [2]. In addition to stromal cells, which actively contribute to all stages of tumorigenesis, the extracellular matrix (ECM) and its components play a central role in shaping the unique nature of the PDAC tumor, defined by its desmoplastic, hypoxic, and immunosuppressive environment [3, 4].

The rich and dense desmoplastic stroma of PDAC, primarily composed of cancer‐associated fibroblasts (CAFs), pancreatic stellate cells (PSCs), and ECM, further contributes to the hypoxic and hypovascular nature of the tumor [2]. This unique TME not only promotes cancer progression but also presents significant challenges for therapeutic strategies. Among the stromal components, PSCs, the most abundant fibroblast‐like stromal cells in the PDAC TME, play a particularly pivotal role in shaping tumor progression. Upon activation, PSCs undergo phenotypic changes, transitioning into myofibroblast‐like cells that drive fibrosis, ECM remodeling, and the secretion of tumor‐promoting factors, thereby contributing to the aggressive nature of PDAC [5].

Despite their central role in the PDAC stroma, PSCs should not be conflated with cancer‐associated fibroblasts (CAFs), as these two cell populations, while functionally interconnected, originate from different sources. PSCs are tissue‐resident fibroblast‐like cells that become activated in response to injury or malignancy, whereas CAFs represent a heterogeneous population with multiple cellular origins, including PSCs, bone marrow‐derived mesenchymal stem cells, and epithelial‐to‐mesenchymal transition (EMT)‐derived fibroblasts. Despite their distinct origins, PSCs and CAFs share functional similarities within the TME, including ECM remodeling, fibrosis, and the secretion of growth factors that sustain tumor progression. Both cell types also exhibit high plasticity, dynamically responding to cancer cell‐derived cues and adapting their phenotypes accordingly. Importantly, while PSCs are a major cellular source of CAFs in PDAC, not all CAFs arise from PSCs, emphasizing the need to distinguish between these populations when investigating tumor‐stroma interactions [6]. The functional heterogeneity of CAFs, as extensively reviewed by Biffi and Tuveson, further underscores the complexity of stromal interactions in PDAC [6].

Although PSCs and their products, such as ECM proteins, are well known for contributing to cancer aggressiveness, emerging evidence suggests they may also exhibit tumor‐suppressive functions depending on the context. This dual role highlights the complexity of PSC biology and underscores the need for a cautious and precise approach when targeting them as part of therapeutic strategies.

Regarding the central role of PSCs in PDAC progression, this review addresses the following key questions based on the current literature:

- What are the multifaceted roles of PSCs in the PDAC TME, and how do they influence cancer cells?
- How do PSCs contribute to therapy resistance and aggressive characteristics of PDAC?
- What are the current and emerging strategies to target PSCs and PSC‐derived ECM components, including preclinical and clinical efforts aimed at disrupting their tumor‐promoting functions to improve patient outcomes?
  By addressing these aspects, this review aims to provide a comprehensive understanding of PSCs in PDAC pathobiology and highlight potential therapeutic avenues targeting these critical players.

## 2 An Overview of the PSCs

PSCs, resident fibroblast‐like cells in the pancreas, play a key role in regulating ECM turnover by synthesizing matrix proteins, matrix metalloproteinases (MMP), and MMP inhibitors. PSCs are key players in pancreatic fibrosis and act as crucial components of the pancreatic injury repair mechanism. These cells are mainly located in the basal regions and lobules of the pancreas and represent one of the most abundant stromal cell types [7, 8]. PSCs exist either in quiescent or active states, depending on external stimuli [5, 7]. In their quiescent state, PSCs maintain pancreatic homeostasis by maintaining the normal function of pancreatic follicles and stabilizing pancreatic pressure [7, 8]. However, disruptions to pancreatic homeostasis, caused by various factors such as trauma, inflammation, viral infection, or cancer invasion, activate PSCs, triggering a phenotypic shift [7, 8]. This activation leads to increased cell proliferation and continuous secretion of ECM components, including collagen and laminin, resulting in the acquisition of a myofibroblast‐like phenotype [7]. While this phenotypic change is reversible under normal conditions, prolonged damage causes persistent overactivation of PSCs. These overactivated cells produce excessive ECM components, which eventually disrupt the dynamic balance of collagen metabolism and induce fibrosis [7, 8].

The reciprocal interactions between PCa cells and PSCs have been well documented in both in vitro and in vivo studies. Interestingly, activated PSCs are detected not only in advanced PDAC tumors but also around pancreatic intraepithelial neoplasms (PanINs), which are early pre‐cancerous lesions [5]. Staining for α‐SMA, a marker for activated PSCs, demonstrates the presence of activated PSCs surrounding PanIN lesions [9]. This observation indicates that PSCs are one of the key players in the progression of PDAC tumors.

PDAC cells actively modulate the behaviors of PSCs by secreting growth factors such as platelet‐derived growth factor (PDGF), fibroblast growth factor 2 (FGF2), and transforming growth factor beta 1 (TGF‐β1), which stimulate further proliferation of PSCs and ECM synthesis [10]. Additionally, PDAC cells enhance PSC‐derived MMP secretion, facilitating ECM turnover, primarily through increased expression of extracellular matrix metalloproteinase inducer (EMMPRIN) and TGF‐β1 signaling [11, 12, 13]. Emerging evidence reveals that PSCs consist of diverse subsets with distinct roles contributing to all stages of PDAC progression [14]. Stromal signaling is essential for PDAC development, as demonstrated by Sherman et al., who showed that KRAS mutations alone are insufficient to drive oncogenic transcription without persistent stromal input [15]. These findings highlight the critical role of PSCs even in the early phases of tumorigenesis. Beyond their contribution to the transition from premalignant to malignant states, PSCs play a key role in promoting and sustaining the aggressive characteristics of PDAC cells, such as induction of epithelial‐mesenchymal transition (EMT), a critical process for cancer cell invasion and migration [16, 17]. In addition to promoting tumor growth and metastasis, PSCs contribute to therapeutic resistance in PDAC [18]. As an example, conditioned media from human PSCs were shown to inhibit the cytotoxic and apoptotic effects of gemcitabine in PANC‐1 cells via stromal cell‐derived factor 1 (SDF‐1α)/C‐X‐C chemokine receptor type 4 (CXCR4) axis [19], which activates mitogen‐activated protein kinase (MAPK) and Phosphoinositide 3‐kinase (PI3K) signaling and increases interleukin‐6 (IL‐6) production, ultimately enhancing chemoresistance. Consistently, PSCs have been found to survive following chemoradiation treatment and exhibit an even more activated phenotype post‐treatment [20].

In vivo studies provide further evidence for the role of PSCs in PDAC progression. Co‐injection of PDAC cells with PSCs resulted in larger tumors with pronounced desmoplasia compared to PDAC cells injected alone, as demonstrated in subcutaneous xenograft [10] and orthotopic mouse models [20, 21]. Moreover, PSCs also facilitate metastasis, as they travel with cancer cells to distant sites, aiding in metastatic seeding and growth [22, 23]. Interestingly, PSCs have been detected in metastatic nodules, suggesting their ability to enter or exit blood vessels, circulate through the bloodstream, and establish microenvironments in distant organs that favor metastasis [22].

The critical roles of PSCs in promoting and sustaining PDAC aggressiveness make them a prominent potential target for effective cancer treatments. However, studies aimed at depleting PSCs or targeting ECM components primarily produced by PSCs have generally yielded disappointing results, as discussed in the following sections. This has led to the suggestion that the increased fibrosis resulting from PSC activation may act as a barrier, limiting the metastasis of cancer cells. Consequently, PSCs exhibit a multifaceted role in PDAC progression. A deeper understanding of the complex crosstalk between PSCs and PDAC cells is crucial for developing effective targeted therapies to disrupt these interactions and improve patient outcomes.

## 3 The Role of PSCs in PDAC Progression and Aggressiveness

**FIGURE 1:**
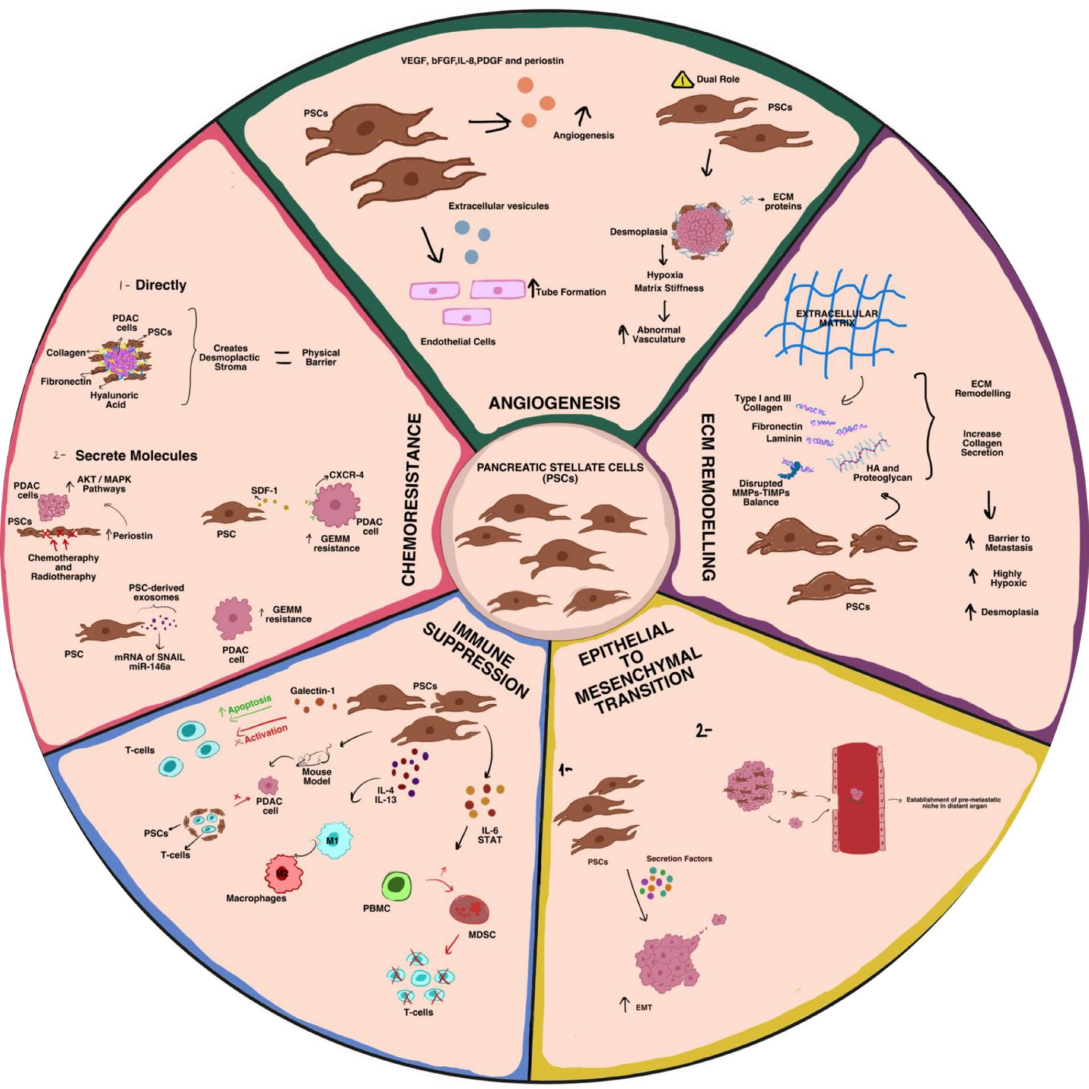
Summary of the role of PSCs in various stages of tumor progression and cancer aggressiveness, highlighting their contributions to chemoresistance, immunosuppression, metastasis, and angiogenesis.

In this section, we focused on the role of PSCs and ECM components produced by PSCs in driving the aggressive nature of PCa, including metastasis, angiogenesis, therapy resistance, and immune suppression. These roles are summarized and illustrated in Figure 1.

### 3.1 PSCs In Remodeling of Stroma

The ECM is a dynamic structure that surrounds cells and regulates various essential cellular processes, including proliferation, migration, and differentiation. It plays a pivotal role in maintaining the structural and functional integrity of tissues [24, 25]. However, excessive ECM remodeling, characterized by changes in composition, abundance, and stiffness, significantly contributes to disease progression, particularly fibrosis in chronic pancreatitis and PDAC [26].

In response to pancreatic injury, PSCs become activated, leading to the overproduction of ECM components such as laminin, fibronectin, and type I and III collagen. Under physiological conditions, type I and III collagen fibrils provide structural integrity and stiffness to the ECM [27]. Crosslinking of collagen fibers, catalyzed by lysyl‐oxidase (LOX) enzymes, is a critical factor in fibril assembly [28]. In PDAC, LOX enzymes are overexpressed, leading to increased collagen crosslinking, which stiffens the ECM and alters cellular migration and invasion and enhances resistance to therapies [29, 30]. Fibronectin, another key ECM protein, amplifies ECM synthesis by binding to latent TGF‐β‐binding protein, facilitating the release of active TGF‐β, which further activates PSCs [31]. Laminins, integral components of the basement membrane, are similarly overproduced, disrupting the surrounding architecture [32]. Laminin 5, composed of α3, β3, and γ2 subunits, promotes proliferation, apoptosis resistance, invasion, migration, and epithelial‐to‐mesenchymal transition [33, 34]. Laminin 5 interacts with cells via focal adhesions and hemidesmosomes, specifically through α3β1 and α6β4 integrins and is negatively associated with patient survival [35]. Hyaluronic acid (HA), a non‐sulfated glycosaminoglycan, is another overexpressed ECM component in PDAC, known for its viscoelastic properties and role in water uptake [36, 37]. HA interacts with the CD44 receptor, activating intracellular signaling pathways such as PI3K‐AKT, ERK, RhoA, and RAS, thereby promoting cell survival, invasion, and EMT [38, 39]. Besides, high levels of HA and collagen I in neoplastic tissues are associated with poor survival outcomes.

MMPs, zinc‐dependent endopeptidases, play a crucial role in ECM degradation, facilitating tumor cell migration, invasion, and metastasis. Specific MMPs, including MMP‐2, MMP‐7, MMP‐9, and MMP‐14, are overexpressed in PDAC. MMP‐2, secreted by activated fibroblasts, activates MMP‐14 at tumor cell filopodia, degrading the basement membrane and promoting cell extravasation [40]. Both MMP‐2 and MMP‐14 can cleave laminin 5, exposing integrin‐binding domains that enhance cell migration and invasion. Moreover, MMP‐7 deficiency in Kras‐driven PDAC mouse models has been associated with smaller tumor mass and reduced metastasis, highlighting its role in disease progression [41].

The dense ECM in PDAC acts as a physical barrier, limiting drug penetration and reducing the efficacy of chemotherapeutic agents as we discussed below [42]. Beyond this physical barrier, the complex tumor‐stroma network contributes to peritoneal spread, vascular invasion, and lymph node metastasis [43]. Interestingly, PSCs have been identified in metastatic nodules, suggesting their ability to circulate and establish supportive microenvironments in distant organs [22]. Furthermore, the desmoplastic stroma of PDAC creates a hypoxic microenvironment due to poor oxygen perfusion. Hypoxia not only supports cancer cell survival but also shifts PSC function from tumor‐suppressive to tumor‐promoting roles. PSCs respond to hypoxia by secreting vascular endothelial growth factor (VEGF) to enhance neovascularization, potentially facilitating metastasis. However, their excessive ECM production exacerbates fibrosis and restricts perfusion [44]. Hypoxic conditions have been shown to enhance PSC‐mediated cancer cell invasion, potentially driven by cancer cells migrating toward better‐perfused regions, which promotes metastasis [45]. Therefore, understanding the roles of ECM components produced by various stromal cells, including PSCs, poses a great importance for the development of effective treatment strategies.

### 3.2 PSC‐Mediated Metastasis

The interaction between PSCs and PDAC cells within the TME plays a pivotal role in ECM remodeling, including increased thickness, altered arrangement of collagen fibrils, and matrix contraction [46]. While ECM stiffness and remodeling are crucial for PSC activation, activated PSCs, in turn, significantly contribute to metastatic processes in PDAC [47, 48]. In this complex and dynamic environment, PSCs primarily enhance the invasiveness and metastatic potential of PDAC cells by inducing EMT [49]. For instance, co‐culture of PSCs with two different PCa cell lines demonstrated reduced cell–cell contact and a shift to fibroblast‐like morphology in cancer cells. This alteration was accompanied by decreased expression of epithelial markers such as E‐cadherin, cytokeratin 19, and membrane‐associated β‐catenin, along with increased expression of mesenchymal markers, including vimentin and Snail (Snai‐1), indicating the induction of PSC‐mediated EMT [16]. Additionally, PSCs have been shown to produce collagen type V, which promotes migration and viability, even in the presence of chemotherapeutic drugs. The effects of collagen V were mediated via the integrin/focal adhesion kinase (FAK) signaling pathway, as inhibition of integrin β1 in PSCs abolished these effects. Consistent with in vitro results, knockdown of collagen V in PSCs reduced metastasis in orthotopic PDAC mouse models [49]. Another study highlighted the role of PSC‐derived hepatocyte growth factor (HGF) in promoting cancer cell invasion and migration via the HGF/c‐Met/survivin pathway [50].

Interestingly, PSCs not only enhance the metastatic capacity of PDAC cells but also actively accompany them to metastatic sites. In vitro and in vivo studies have shown that PSCs facilitate cancer cell migration and travel alongside circulating tumor cells (CTCs) to distant organs [22, 51]. Zhang et al. showed that cancer cell‐derived exosomes recruit PSCs from the bloodstream, ensuring that migrating cancer cells bring supportive stromal components to establish a favorable metastatic niche [52]. Supporting this, human PDAC tissues with lymph node metastases exhibited higher stromal immunoreactivity for α‐SMA, desmin, and MMP2, markers for PSCs, compared to non‐metastatic tissues [53]. Surprisingly, PSCs are active participants even in the early stages of metastasis, collaborating closely with PDAC cells [23]. Beyond hematogenous metastasis, PSCs also facilitate metastasis via nerves. A recent study showed that PSCs can facilitate the perineural invasion of cancer cells, an alternative route for metastasis, through activation of the HGF/c‐met pathway [54].

Although many aspects of the mechanistic role of PSCs in the metastatic process remain unknown, it is clear that these cells are important players in both local and distant metastasis in PDAC. This highlights the urgent need for further studies to unravel the underlying mechanisms and to develop targeted strategies aimed at disrupting PSC‐mediated metastasis.

### 3.3 PSCs‐Mediated Angiogenesis

Angiogenesis, the formation of new blood vessels, is an essential process for the growth and survival of solid tumors. Rapidly proliferating cells require an increased supply of nutrients and the efficient removal of cellular waste. Without angiogenesis, tumors cannot grow beyond 1–2 mm in diameter [55].

While PSCs are well recognized for their role in secreting large amounts of ECM proteins and creating a fibrotic stroma, they also produce various pro‐angiogenic factors, including VEGF, bFGF, IL‐8, PDGF, and periostin [56]. Both in vitro and in vivo studies demonstrate that PSCs exhibit critical roles in promoting angiogenesis. As an example, TGF‐β secreted by PSCs was reported to support tube formation in endothelial cells during angiogenesis [57]. Similarly, PSCs were found to stimulate endothelial cell proliferation and tube formation via the HGF/c‐Met pathway in vitro [58]. In addition, PSC‐derived MMP9 facilitates the early stages of angiogenesis by degrading the basement membrane, thereby enabling endothelial cell invasion [59] Moreover, the synergistic interaction between PSCs and PDAC cells can further induce angiogenesis. For instance, PDAC‐derived cytokines, such as TGF‐β1 and PDGF, stimulate PSCs to produce prokineticin, a secreted protein involved in regulating various biological processes, including angiogenesis [60].

Hypoxia, often driven by overactivated PSCs, further amplifies their pro‐angiogenic role. Under low oxygen tension, PSCs exhibit increased expression of type I collagen and VEGF, which are essential for angiogenesis under hypoxic conditions [61]. The last, but not the least, matrix stiffness, a hallmark of PSC‐mediated desmoplasia, also plays a critical role in regulating angiogenesis. Increased ECM stiffness can alter cell behaviors such as cell–cell adhesion, migration, and invasion, which are essential for angiogenesis. For instance, endothelial cell invasion is induced by matrix stiffness, which is maintained by upregulation of MMPs and increased N‐cadherin levels [62].

Interestingly, PSCs may also act as regulators of angiogenesis by balancing pro‐angiogenic and anti‐angiogenic factors. While they are known to release pro‐angiogenic molecules, PSCs are also reported to secrete anti‐angiogenic molecules, such as thrombospondin‐1 [63], vasohibin‐1, and endostatin [64]. This dual role of PSCs in angiogenesis suggests that they may contribute differently to various stages of PDAC progression, depending on the specific conditions within the TME. In conclusion, the role of PSCs in angiogenesis appears to be context‐dependent. Depletion of PSCs in the PDAC TME has been shown to suppress angiogenesis and exacerbate hypoxia without improving the efficacy of gemcitabine [65]. These findings suggest that rather than the complete depletion of PSCs, strategies aimed at normalizing PSC activity and tumor vasculature may provide a more effective and safer therapeutic approach.

### 3.4 The Role of PSCs in Therapy Resistance

#### 3.4.1 PSCs‐Mediated Chemoresistance

The dense desmoplastic stroma in PDAC acts as a physical barrier, significantly limiting the efficacy of chemotherapeutic drugs. This has been evidenced by increased intratumoral drug delivery and penetration following stromal depletion treatments [66]. Activated PSCs were shown to produce substantial amounts of ECM components, such as collagen I/III, laminin, desmin, and fibronectin [67], which lead to fibrosis and reduced vascularity, further hindering delivery of drugs to tumor cells.

Additionally, PSCs contribute to chemotherapy resistance by inducing stem cell‐like phenotypes in cancer cells [68]. In a study, PSCs were shown to create a supportive paracrine niche for pancreatic cancer stem cells (CSCs) by secreting Nodal/Activin, which promotes sphere formation in vitro [69]. Hypoxia, mediated by PSC activity, indirectly enhances the CSC phenotype. PSC‐induced ECM‐mediated hypoxic conditions have been shown to drive EMT in both CSCs and cancer cells, promoting their invasive phenotype [70]. Recently, PSC‐derived TGF‐β1 was reported to negatively regulate the cell adhesion molecule L1CAM, harboring enhanced stemness potential and tumorigenicity [71]. Additionally, an in vivo study demonstrated that PSCs can uptake and metabolize gemcitabine, thereby reducing its availability to PDAC cells [72]. This scavenging effect is attributed to the low expression of inactivating enzymes in PSCs, allowing them to sequester the drug within the tumor stroma. Furthermore, TGF‐β‐activated PSCs express cysteine‐rich angiogenic inducer 61 (CYR61), a matricellular protein that regulates the nucleoside transporters hENT1 and hCNT3, responsible for the cellular uptake of gemcitabine [73]. This mechanism helps to deprive tumor cells of gemcitabine, leading to treatment failure.

PSCs secrete various molecules that interact with cancer cells, further contributing to drug resistance [74]. For instance, periostin, a marker of activated PSCs, plays a role in regulating ECM production by promoting collagen crosslinking and stability. Beyond its role in forming a physical barrier that impairs drug delivery, periostin has been demonstrated to protect cancer cells from chemotherapy and radiotherapy through paracrine signaling and activation of the AKT and MAPK pathways [75]. Similarly, another study reported that periostin enhances gemcitabine resistance and promotes cancer cell invasiveness via the ERK1/2 and FAK/AKT pathways [76]. Other PSC‐secreted factors, such as SDF‐1, have been reported to bind to the CXCR4 receptor on cancer cells, increasing their resistance to gemcitabine [77]. Additionally, PSC‐derived hepatocyte growth factor (HGF) has been shown to increase gemcitabine resistance via the c‐Met/PI3K/AKT pathway [78], while fibronectin induces resistance through the ERK 1/2 pathway [79]. Furthermore, PSC‐derived exosomes also contribute to gemcitabine resistance by transmitting Snail mRNA and an oncogenic microRNA, miR‐146a [80].

PSCs are also implicated in radiotherapy resistance. Mantoni et al. demonstrated that PSCs exert a radioprotective effect on PDAC cells through Integrin‐β1 signaling [81]. Additionally, the presence of PSCs in the culture environment has been reported to help protect PCa cells from radiation‐induced cell death [19, 82, 83]. In summary, these findings underscore the critical role of PSCs in mediating chemo‐ and radio‐resistance, either via ECM remodeling or through the secretion of signaling molecules. To overcome this resistance, simultaneous inhibition of PSC‐mediated signaling pathways or the development of targeted therapies to prevent PSC activation is crucial in improving treatment outcomes.

#### 3.4.2 Immunosuppressive Role of PSCs in PDAC

Enhancing anti‐tumor immunity and the efficacy of immunotherapy remains a significant challenge in PDAC treatment. The tumor‐mediated immunosuppressive microenvironment of PDAC plays a critical role in immune evasion and T cell dysfunction. In addition to cancer cells, this suppression is largely driven by the infiltration of suppressive immune cells and stromal components, particularly PSCs [46]. PSCs are known to exert diverse immunosuppressive effects, complicating therapeutic interventions. Notably, α‐SMA+ and FAP+ subtypes of PSCs are key contributors to ECM production in the PDAC TME. Fibroblast activation protein α (FAP‐α) interferes with anti‐tumor immunity, as evidenced by a subcutaneous mouse model of PDAC where depletion of FAP‐α induced an immune response and resulted in tumor regression [84].

PSCs secrete various soluble cytokines that contribute to T cell exhaustion and dysfunction. For example, PSC‐derived CXCL12 (also known as SDF‐1) limits the activity of cytotoxic T cells and promotes the differentiation of macrophages into a pro‐tumorigenic M2 phenotype. CXCL12 also recruits myeloid‐derived suppressor cells (MDSCs) and tumor‐associated neutrophils to the tumor to further create an immunosuppressive environment [85]. Furthermore, PSC‐derived interleukins, such as IL‐4 and IL‐13, drive macrophage polarization toward the M2 phenotype, which, in turn, activates PSCs via PDGF and TGF‐β signaling, thereby creating a vicious cycle [86]. The accumulation of MDSCs in the PDAC TME is correlated with poor prognosis [87, 88]. PSCs not only recruit MDSCs into the tumor but also enhance their differentiation and expansion through the IL‐6/STAT‐3 pathway [89]. Besides, PSC‐derived IL‐6 and STAT‐3 signaling have been shown to induce the differentiation of peripheral blood mononuclear cells (PBMCs) into MDSCs while simultaneously inhibiting T cell proliferation [90]. Additional immunosuppressive cytokines secreted by PSCs, such as IL‐10, TGF‐β, VEGF, monocyte chemoattractant protein 1 (MCP‐1), Granulocyte‐macrophage colony‐stimulating factor (GM‐CSF), and prostaglandin E2 (PGE2), also contribute to immune evasion and impaired anti‐tumor responses [91]. Additionally, PSC‐mediated desmoplasia restricts cytotoxic T cell infiltration [92, 93]. For instance, PSC‐secreted galectin‐1 was shown to inhibit T cell activation and induce their apoptosis, shifting T cells toward a T helper type 2 (Th2) phenotype, thereby creating an immunosuppressive environment [94].

Another critical mediator of immune regulation is FAK1, a tyrosine kinase that regulates T cell survival, cytokine production, and migration. FAK1 is elevated in PDAC tumors and associated with increased fibrosis and reduced CD8+ T cell accumulation. In a mouse model study, PSCs were shown to inhibit the CD8+ T cell migration to the stromal peritumoral compartments, thereby preventing these immune cells from directly accessing PDAC cells [95]. Additionally, ECM components secreted by PSCs, such as collagen and fibronectin, activate Rho kinase‐dependent FAK1 signaling, further suppressing anti‐tumor immunity [96]. The hypoxic and acidic microenvironment in PDAC, primarly driven by PSCs, also impairs immune responses. Hypoxia‐inducible factor 1‐alpha (HIF‐1α) activation has been shown to suppress immunity and promote tumor immune evasion, although the underlying mechanisms require further investigation [97, 98].

Overall, PSCs are potent immunosuppressive modulators within the PDAC TME, acting through multiple mechanisms, including the secretion of immunomodulatory cytokines and chemokines and the establishment of a dense, desmoplastic stroma. However, there remain numerous unresolved mechanistic points to identify the role of PSCs in immune regulation in PDAC, necessitating further research.

## 4 Several Approaches for Targeting Pancreatic Cancer Tumor Stroma

**FIGURE 2:**
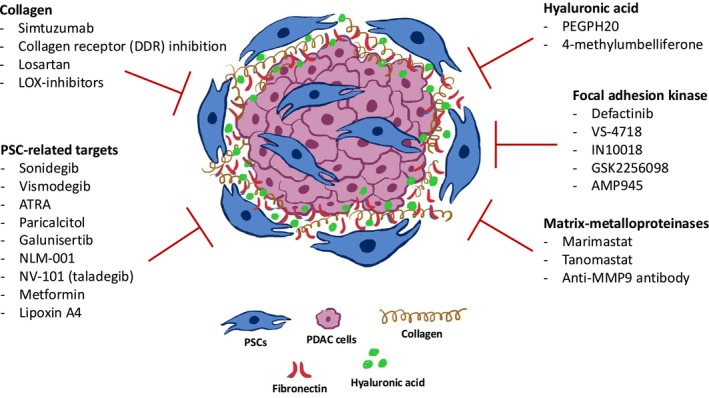
Examples of therapeutic approaches targeting PSCs or ECM components, evaluated in preclinical and/or clinical studies, aimed at disrupting the tumor microenvironment to enhance treatment efficacy.

The TME in PDAC is characterized by a dense ECM composed of collagen, hyaluronic acid, fibronectin, elastin, and other sulfated glycosaminoglycans. PSCs, a cell type unique to the PDAC TME, are the primary source of the dense fibrotic stroma observed in PDAC tissues. This chapter focuses on PSC‐mediated therapeutic approaches and highlights the therapeutic potential of key ECM components by reviewing recent advancements in the literature. Some of the ECM targets evaluated in preclinical and clinical settings are illustrated and summarized in Figure 2.

### 4.1 Strategies for Targeting PSCs

The unique and dense microenvironment of PDAC is one of its defining characteristics and a significant contributor to treatment failure. While stromal cells exhibit anti‐tumorigenic properties in the early stages of tumor progression, they become tumor‐supportive as the disease progresses. Since most chemotherapeutics target rapidly proliferating tumor cells, stromal cells often remain unaffected by therapy, creating a protective and supportive niche for surviving cancer cells. Consequently, targeting both stromal and cancer cells has gained increasing attention in recent years. This section specifically focuses on the recent therapeutic approaches aimed at targeting or reprogramming PSCs, the most dominant stromal cell type in the PDAC TME.

As detailed above, PSCs play a central role in producing fibrotic stroma and actively contribute to tumor progression and cancer aggressiveness, making them a key focus in TME‐targeting approaches. However, this strategy is not as straightforward as it may seem and can sometimes yield unexpected outcomes. For instance, Ozdemir et al. demonstrated that deleting α‐SMA^(+)^ myofibroblasts in transgenic mice resulted in invasive, undifferentiated tumors with enhanced hypoxia and resistance to gemcitabine, while anti‐CTLA4 immunotherapy effectively reversed disease progression [98]. Similarly, another study using genetically engineered mouse models (GEMMs) demonstrated that genetic depletion of Sonic Hedgehog (Shh) signaling reduced the fibrotic barrier. However, this also led to increased tumor invasiveness and metastasis, with the remaining stromal cells likely shifting toward a more pro‐tumorigenic phenotype, such as inflammatory cancer‐associated fibroblasts (iCAFs) [99]. These results underscore the need for caution when targeting stromal cells in PDAC. Therefore, most studies have shifted their focus to targeting stromal‐cancer cell interactions or reprogramming PSCs rather than directly eliminating them. As discussed below, numerous preclinical studies and a few clinical trials have explored strategies for targeting or reprogramming PSCs.

Recent preclinical studies have revealed promising strategies for reprogramming PSCs or inducing a quiescent phenotype [9]. Our previous work demonstrated that suppressing periostin—a protein produced by PSCs that regulates collagen crosslinking—reduced extracellular collagen density and increased NK cell infiltration into 3D PDAC spheroids [100]. Similarly, in orthotopic PDAC mouse models, inhibiting HGF and c‐Met reduced disease progression following resection, displayed anti‐angiogenic effects, and decreased the number of circulating active PSCs [101]. Additionally, the endogenous bioactive lipid, Lipoxin A4 (LXA4), was shown to inhibit TGF‐β mediated differentiation of PSCs and reduced tumor growth with a significant reduction in collagen1 expression and fibrosis [9]. Furthermore, Jolly et al. demonstrated that cholecystokinin (CCK) receptor antagonists induce a quiescent state in PSCs, thereby decreasing fibrosis [102]. More recently, a 2023 study highlighted the role of mechanosensing through N‐cadherin in reprogramming activated PSCs into a quiescent state. The use of an N‐cadherin‐mimicking peptide successfully reversed PSC activation and restored quiescence [103].

Other therapeutic approaches have explored repurposing existing drugs. For instance, mebendazole, an antiparasitic drug, reduced PSC activity and desmoplasia, decreasing tumor size in two different GEMMs of PDAC [104]. Similarly, metformin, an anti‐diabetic drug, inhibited fibrogenic cytokine production and PSC activation in PDAC xenograft models, resulting in lower stromal density, improved drug infiltration, and enhanced gemcitabine efficacy [105]. In the other study, calcipotriol, a vitamin D receptor ligand used for plaque psoriasis, was shown to significantly reduce tumor volume, increase gemcitabine penetration, and extend mouse survival by 57% when combined with gemcitabine compared to chemotherapy alone [63]. Likewise, all‐trans retinoic acid (ATRA), used for acute promyelocytic leukemia treatment, reduced stroma reactivity by inducing PSC quiescence and decreased cancer cell proliferation in vitro and in vivo [106]. Additionally, inhibiting autophagy in PSCs using chloroquine, an antiparasitic drug, or small interfering RNAs increased lipid droplet accumulation in PSCs, indicating a shift toward quiescence, and reduced ECM production, thereby resulting in smaller tumor formation and fewer metastases in mouse models [107]. Metformin was also shown to suppress TGF‐ß signaling, as well as collagen and hyaluronic acid production in PSCs in vitro. Consistently, metformin reduced inflammatory cytokines, M2 pro‐tumorigenic macrophage polarization, and desmoplasia by limiting ECM remodeling [108].

Innovative combination therapies have further advanced PSC‐targeting strategies. In a study, a tumor microenvironment‐responsive nanosystem was developed using PEGylated polyethylenimine‐coated gold nanoparticles for the co‐delivery of ATRA and siRNA targeting heat shock protein 47 (HSP47). This approach re‐educated PSCs into a quiescent state, inhibited ECM hyperplasia, and improved the delivery of gemcitabine [109]. Advanced nanoformulation‐based approaches have also shown promise. A 2022 study demonstrated that co‐delivering TNF‐Related Apoptosis Inducing Ligand (TRAIL) and nitric oxide (which prevents tissue desmoplasia) via a nanoformulation effectively targeted both tumor cells and PSCs in orthotopic PDAC mouse models. Nitric oxide reprogrammed active PSCs, reduced desmoplasia, and enhanced the penetration and efficacy of TRAIL [110]. Similarly, the combination of a nanoformulation of chloroquine with gemcitabine decreased the density of activated PSCs and reduced tumor progression in a PDAC xenograft mouse model [111].

However, it is crucial to acknowledge that these targeting strategies may also impact other cells within the TME, leading to a reduction in the stromal compartment. To overcome this limitation, more sophisticated nanotechnology‐based drug delivery systems have recently been developed and tested to achieve precise targeting of PSCs. For example, the nanoformulation of ATRA‐loaded liposomes containing calcium phosphate cores and arginine 12‐mer peptide (r12) ligands, shielded by fibroblast activation protein (FAP)‐detachable polyglutamate, was designed and evaluated in PSCs [112]. Another study tested a multifunctional nanoparticle—tripeptide RFC‐modified gelatin/oleic acid nanoparticle (RNP@ATRA)—, which delivers ATRA in an enzyme‐triggered, popcorn‐like manner in in vivo mouse models [113]. Similarly, an ATG5 siRNA‐containing nanoparticle formulation, composed of cancer cell membrane‐fused liposomes, was designed to specifically target PSCs [114]. These nano‐formulations demonstrated enhanced in vivo internalization by PSCs, induced PSC quiescence, and disrupted stromal barriers, ultimately improving intratumoral drug delivery. Furthermore, an innovative liposomal system, MR‐T‐PD, was developed to simultaneously target tumor cells and activated PSCs. This smart formulation facilitated tumor‐specific activation, enabling the controlled release of doxorubicin to eliminate cancer cells and calcipotriol to suppress aPSCs. As a result, MR‐T‐PD significantly reduced tumor growth while minimizing side effects [115].

Additionally, chemophotothermal therapy has emerged as a novel strategy. In a recent study, C‐G nanoparticles were designed to carry acid‐responsive photothermal molecules and gemcitabine [116]. The nanoparticles deactivated PSCs, reduced TGF‐β production, and collagen fiber expression. Moreover, hyperthermia induced by photothermal therapy remodeled the ECM, improved nanoparticle penetration, and enhanced therapeutic efficacy by downregulating key genes involved in tumor progression [116].

Despite promising preclinical results, the clinical translation of PSC‐targeting strategies remains limited. A phase Ib trial repurposed ATRA as a stromal modulator for PDAC treatment (NCT03307148). In this study, ATRA was combined with chemotherapy in 27 patients with unresectable PDAC. The combination of ATRA with gemcitabine and nab‐paclitaxel was found to be safe and tolerable. Its impact on survival in locally advanced PDAC patients will be evaluated in a phase II randomized controlled trial (NCT04241276). Another study that investigated the combination of gemcitabine with calcipotriol was shown to induce PSC quiescence and reduce inflammation and fibrosis in a KPC mouse model of PDAC [63]. Based on these findings, a clinical trial evaluating this combination is ongoing (NCT03520790).

In summary, although research on PSC‐targeting strategies continues to expand, effectively eliminating or reprogramming these cells remains a complex challenge. As discussed by Yuan and colleagues, cellular plasticity is a critical factor enabling cancer cells to adapt and survive under extreme conditions [117]. This plasticity extends beyond cancer cells to the stromal compartment, further contributing to tumor aggressiveness. PSCs, particularly CAFs, exist in distinct subtypes with different functions, including myofibroblastic CAFs (myCAFs), inflammatory CAFs (iCAFs), and antigen‐presenting CAFs (ap‐CAFs) [118, 119]. However, the precise origins of these subpopulations—such as whether PSCs differentiate into specific CAF subtypes—remain unclear. This uncertainty makes the selective targeting of PSC‐derived subpopulations one of the most complex and challenging therapeutic strategies for PDAC.

### 4.2 ECM‐Targeting Approaches

#### 4.2.1 Hyaluronic Acid

Hyaluronic acid (HA), a glycosaminoglycan, is a crucial component of ECM in both homeostasis and disease. HA is predominantly produced by tumor and stromal cells, and its molecular weight determines its function, influencing whether it plays a pro‐tumorigenic or anti‐tumorigenic role. High molecular weight HA is primarily found under homeostatic conditions and exhibits anti‐inflammatory and anti‐proliferative properties. Conversely, low molecular weight HA is mainly observed in tumor tissues. During tumor progression, HA is degraded by hyaluronidases into small fragments, which exhibit pro‐inflammatory and pro‐angiogenic properties, thereby promoting tumor aggressiveness [120]. In addition to molecular weight, the abundance of HA is significant as it contributes to the formation of dense stroma and represents a poor prognostic factor for PDAC [121]. Therefore, hyaluronic acid represents an important therapeutic target for pancreatic cancer. Three main approaches exist to target HA, which are inhibition of HA synthesis, blocking HA signaling, and depleting stromal HA.

In one of the early studies investigating the inhibition of HA synthase, the administration of 4‐methylumbelliferone (4‐MU), a drug that inhibits HA synthases, was shown to enhance the anticancer activity of gemcitabine in PDAC cell lines [122]. However, the lack of stromal components in the cell culture model was a significant limitation of this study. Therefore, more recently, the effect of 4‐MU was evaluated in a co‐culture model of PDAC cells with fibroblasts to better mimic the TME. The co‐culture of PDAC cells with fibroblasts increased HA production, as evidenced by elevated HA synthase 3 (HAS3) mRNA levels. Besides, treatment with 4‐MU notably reduced HA production and the migration of cancer cells [123]. The results of in vivo experiments were also consistent with in vitro findings. 4‐MU treatment was shown to suppress HA accumulation in PDAC tumors and improve the survival period of the mice [124].

After promising preclinical study results, clinical studies targeting HA were conducted. A phase Ib/II clinical trial evaluated PEGPH20, a PEGylated human hyaluronidase, combined with modified FOLFIRINOX (mFOLFIRINOX) in 138 untreated metastatic PDAC patients. Surprisingly, the median overall survival (OS) was nearly half in the combination group vs. chemotherapy alone (7.7 months vs. 14.4 months) [125]. Adverse effects, on the other hand, occurred in 45% of patients treated with combination therapy, while it was 9% in the chemotherapy alone group. Although the OS results were discouraging, four of 55 patients receiving combination therapy had a complete response, an exceptionally rare outcome in advanced PDAC. In another study, a phase III randomized controlled trial (HALO‐109‐301), metastatic PDAC patients were treated with gemcitabine plus nab‐paclitaxel (AG) with or without PEGPH20 [NCT02715804, 126]. The study was terminated due to toxicity and serious adverse events, with no OS improvement (11.2 vs. 11.5 months).

To date, six clinical trials involving HA‐targeting therapies have been registered on ClinicalTrials.gov. Despite some promising outcomes, as summarized above, three of the studies were terminated (due to safety concerns, sponsor decisions, or halted enrollment). Currently, one active study is listed on ClinicalTrials.gov, investigating the effect of PEGPH20 in combination with nab‐paclitaxel and rivaroxaban, an anticoagulant drug, in patients with advanced PDAC (NCT02921022).

#### 4.2.2 Collagen

Collagen is the most abundant ECM component in the stroma of PDAC, making it a promising therapeutic target for stroma‐targeting treatment approaches. As a result, numerous studies have focused on strategies such as targeting collagen production, disrupting collagen fibers, and inhibiting collagen‐mediated signaling, among other approaches. For example, the use of an antifibrotic agent, halofuginone, demonstrated a reduction in the stromal barrier in GEMM of PDAC [127]. The results showed that halofuginone decreased fibroblast activity, which in turn reduced the deposition of ECM components, including collagen and hyaluronic acid. Additionally, halofuginone treatment improved the delivery of chemotherapeutics and altered the immune profile, increasing the infiltration of classically activated inflammatory macrophages and cytotoxic T cells, which further caused intratumoral necrosis and a reduction in tumor volume [127]. Similarly, the angiotensin receptor II inhibitor, losartan, was shown to inhibit collagen I production in CAFs, thereby reducing the desmoplastic response in several cancer types, including pancreatic cancer [128]. In a phase II clinical trial, treatment of unresectable PDAC patients with losartan was reported to improve the delivery of therapeutic nanoparticles and chemotherapy, as well as enhance margin‐negative resection rates [129]. In addition, a recent study tested a collagenase‐encapsulated nanoparticle system, known as “collagozome,” designed to directly target collagen. The results revealed that collagozome pre‐treatment reduced fibrotic tissue, increased drug penetration into the pancreas, and decreased tumor volume in mice [130].

Disruption of collagen crosslinking and stabilization to reduce stroma density is another emerging strategy for targeting non‐cellular compartments of the stroma. Simtuzumab, an antibody targeting the enzyme involved in collagen crosslinking, LOXL2, was tested in the clinical setting for its efficacy and safety. A phase III trial of simtuzumab in combination with gemcitabine was conducted in patients with metastatic PDAC (NCT01472198). However, no significant improvement was observed regarding progression‐free survival (PFS), overall survival (OS), or objective response rate (ORR) compared to gemcitabine alone [131].

Targeting collagen‐mediated signaling rather than directly targeting collagen itself, might offer an alternative approach. For instance, the triple‐helical structure of collagen binds to cell surface receptor tyrosine kinases, specifically Discoidin domain receptor (DDR1/2), which in turn promotes cancer cell survival and tumor progression, indicating that it might be a therapeutic target. Pharmacologic inhibition of DDR1 using a small‐molecule inhibitor has demonstrated promising results, including reduced colony formation and migration of PCa cells. Moreover, this inhibition was shown to reduce tumor burden and enhance response to chemotherapy in orthotopic xenograft models [132].

Interestingly, both cancer cells and other stromal cells may be responsible for the production of different forms of collagen. In one study, fibroblasts were found to produce type 1 collagen (Col1) heterotrimers (α1/α2/α1), while PCa cells specifically produce an abnormal Col1 homotrimer (α1/α1/α1). The study demonstrated that deletion of the Col1 homotrimer led to enhanced T cell infiltration and improved efficacy of anti‐PD‐1 immunotherapy [132]. Similarly, another study used dual‐recombinase genetic mouse models of PDAC to delete Col1 specifically in αSMA^+^ myofibroblasts (PSCs). A significant reduction in stromal Col1 led to upregulation of Cxcl5, which is associated with the recruitment of myeloid‐derived suppressor cells and suppression of cytotoxic T cells. Additionally, Col1 deletion accelerated the emergence of PanINs and PDAC, resulting in decreased overall survival in mice [133]. These findings further underscore the importance of targeting “right” type of collagen to suppress PDAC aggressiveness.

#### 4.2.3 Matrix Metalloproteinases

One of the defining features of the desmoplastic stroma in PDAC is its dynamic alterations, including excessive ECM deposition, remodeling, and degradation [134]. MMPs, a group of enzymes responsible for the remodeling of ECM, are frequently overexpressed in the stroma of PDAC. The proteolysis of ECM components by proteolytic enzymes, such as MMPs, may lead to generating small peptides, which can interact with cancer cells, thereby promoting cancer metastasis and aggressiveness [135]. An example of this phenomenon is the behavior of collagen I, one of the major ECM proteins, which differs depending on whether it remains full length (intact) or is cleaved by MMPs. The cleaved form of collagen I was found to promote PDAC progression by activating the DDR1–NFκβ–NRF2 pathway, whereas intact Collagen I inhibits tumor growth by triggering DDR1 degradation [136]. Consistent with these molecular findings, patient survival data reveal that individuals with intact collagen I have better outcomes compared to those with its cleaved form. Analysis of 106 PDAC patients demonstrated that patients with full‐length collagen I had better survival than those with cleaved collagen, highlighting the potential clinical utility of MMP inhibitors [136].

Preclinical studies further support the therapeutic potential of targeting MMPs. For instance, in one study, an anti‐MMP‐9 antibody was used in combination with nab‐paclitaxel + gemcitabine in mouse models of PDAC. The combination therapy significantly reduced stromal and EMT markers compared to treatment with nab‐paclitaxel and gemcitabine alone. Additionally, the anti‐MMP‐9 antibody decreased tumor burden and improved mouse survival [137].

Despite encouraging preclinical findings, clinical trials of MMP inhibitors in PDAC have yielded limited success. In a phase II randomized trial, marimastat, a broad‐spectrum MMP inhibitor, was tested in unresectable PDAC patients and compared to gemcitabine. The results showed no significant difference in one‐year survival rates between the two groups [138]. Similarly, combining marimastat with gemcitabine did not improve median or one‐year survival rates in unresectable PCa patients compared to gemcitabine alone [139]. Another MMP inhibitor specific to MMP‐2, ‐3, and ‐9, tanomastat, was also evaluated in advanced PDAC patients. Unfortunately, the results revealed that tanomastat was less effective than gemcitabine, as patients treated with tanomastat had shorter median survival compared to those receiving gemcitabine alone [140].

Given the lack of success in directly targeting MMPs in the clinical trials, recent efforts have shifted toward leveraging MMP activity for drug delivery using nanoparticles. For instance, in a study, MMP‐2 responsive liposomes were developed to deliver the antifibrotic drug (pirfenidone) to tumor tissue. This strategy effectively reduced desmoplasia by decreasing collagen I, fibronectin, and tenascin C protein levels, thereby enhancing the penetration of chemotherapy in PDAC xenograft models [141]. In addition, in another study, a collagen mimetic lipopeptide sensitive to MMP‐9 was developed. The particles were found to be stable in physiological conditions, and they were hydrolyzed by MMP‐9 activity and released their drug cargo. The results showed that the nanovesicles were capable of encapsulating gemcitabine with 50% efficiency. Additionally, the effectiveness of the particles was shown in both PCa cell culture and mouse models [142].

#### 4.2.4 Focal Adhesion Kinase (FAK)

FAK, a type of non‐receptor tyrosine kinase, is activated by ECM receptors and plays a significant role in ECM stiffness [143]. Notably, activated FAK was shown to correlate with increased collagen deposition and infiltration of granulocytes, as well as decreased infiltration of cytotoxic T cells [95]. This information underlines the importance of FAK in modulating the TME in PDAC.

In preclinical studies, FAK inhibition has demonstrated promising therapeutic potential. For example, FAK inhibition with defactinib displayed anti‐proliferative and anti‐migratory effects in PDAC cell lines. The combination of defactinib with nab‐paclitaxel exerted a synergistic effect on cell proliferation in vitro and reduced tumor growth in vivo [144]. Similarly, treatment with a small‐molecule FAK inhibitor in KPC mice resulted in a reduction in tumor fibrosis, progression, metastasis, alongside a decrease in immunosuppressive myeloid cell populations, ultimately leading to improved survival [95]. Additionally, inhibition of FAK using a selective FAK inhibitor, VS‐4718, was shown to reduce tumor fibrosis, decrease the number of tumor‐infiltrating immunosuppressive cells, and thereby reduce tumor progression and result in doubling survival in KPC mice [141]. Moreover, administering a FAK inhibitor to KPC mice three days prior to chemotherapy reduced stromal fibrosis and stiffness, and thereby improved penetration of chemotherapy [129].

The combination of FAK inhibition with other therapeutic modalities, including immunotherapy, is an area of growing interest. For instance, a study investigating the efficacy of a FAK inhibitor (FAKi) and G47Δ, a third‐generation oncolytic herpes simplex virus type 1, in combination with or without immune checkpoint inhibitors showed promising results [145]. The combination of FAKi and G47Δ reduced tumor stromal content and reprogrammed the TME to favor immune stimulation and enhanced efficacy in both subcutaneous and orthotopic tumor models. Interestingly, adding immune checkpoint inhibitors to the combination significantly prolonged survival compared to FAKi alone, highlighting the potential of FAK inhibitors to enhance the efficacy of stroma‐targeting therapies in PDAC. Similarly, FAK inhibitor IN10018, when combined with PEGylated liposomal doxorubicin (PLD) demonstrated synergistic effects, increasing tumor‐infiltrating lymphocytes and promoting immunogenic cell death [146].

Clinical trials investigating FAK inhibitors in PDAC are also underway. A phase I, dose escalation study showed that the combination of defactinib with pembrolizumab (immune checkpoint inhibitor and gemcitabine was well tolerated with no dose‐limiting toxicities [NCT02546531, 147]). In a phase II study, the effect of a FAK inhibitor, GSK2256098, in combination with a MEK inhibitor, trametinib, was evaluated in advanced PDAC patients who had progressed after first‐line chemotherapy (NCT02428270). While the combination therapy was found to be safe and well tolerated, it did not result in clinical benefit [148]. Finally, three phase II clinical trials are ongoing, exploring combinations of defactinib with different treatment approaches, such as chemotherapy, immunotherapy, Ras inhibitors, and radiotherapy (NCT03727880, NCT05669482, NCT04331041).

## 5 Clinical Trials for Stroma‐Targeting Strategies

As discussed earlier, numerous preclinical studies and clinical trials have been conducted or are currently ongoing to explore strategies for targeting the PDAC stroma, with the ultimate goal of improving patient quality of life and survival rates. This chapter specifically focuses on summarizing clinical trials aimed at targeting the PDAC stroma. Table 1 provides an overview of completed and terminated studies, along with brief summaries of their outcomes.

**TABLE 1:** Completed or terminated clinical trials for targeting PDAC stroma.

Target	Compound	Combination	Trial phase	Status	Outcome summary	Trial number
Hyaluronic acid	PEGPH20	Nab‐paclitaxel and gemcitabine	II	Completed	Significantly improved PFS of PDAC patients with high HA levels	NCT01839487
		FOLFIRINOX	II	Completed	Median OS of patients was lower in combination arm compared to FOLFIRINOX alone	NCT01959139
		Nab‐paclitaxel and gemcitabine	III	Terminated	Combination did not improve OS and PFS. Grade ≥ 3 adverse events were observed in combination arm	NCT02715804
Collagen	Simtuzumab	Gemcitabine	II	Completed	Combination did not improve clinical outcomes of patients with metastatic PCa	NCT01472198
CAFs/PSCs (Hedgehog pathway)	Sonidegib	Fluorouracil, Leucovorin, Oxaliplatin, Irinotecan	I	Completed	Treatment did not enhance PFS or OS.	NCT01485744
	Vismodegib	Gemcitabine Hydrochloride	II	Completed	Combination was not superior to gemcitabine alone	NCT01195415
	Vismodegib	Gemcitabine	II	Completed	Treatment did not improve overall response rate (ORR), PFS, or OS of patients	NCT01064622
		Gemcitabine and nab‐paclitaxel	II	Completed	Combinational use of vismodegib did not improve efficacy of drugs.	NCT01088815
CAFs	ATRA	Gemcitabine plus nab‐paclitaxel	I	Completed	Combination was found to safe and tolerable and evaluated in phase II trial (NCT04241276)	NCT03307148
	Paricalcitol	Liposomal irinotecan and 5‐FU/Leucovorin	I	Completed	Combination is well tolerated in patients, however does not appear to improve response rate or survival outcomes	NCT03883919
	Paricalcitol	Albumin‐bound paclitaxel Cisplatin Gemsitabine	II	Completed	The treatment resulted in high response rates (84% ORR; complete and partial response)	NCT02754726
FAK kinase	GSK2256098	Trametinib	II	Completed	The treatment was well tolerated but lacked activity in unselected advanced PDAC	NCT02428270
CAFs/PSCs (TGF‐β)	Galunisertib	Gemcitabine	Ib/II	Completed	Combination improved overall survival with minimal added toxicity	NCT01373164

As summarized in Table 1, several approaches have been employed to target HA. The combination of PEGPH20 with chemotherapeutics such as nab‐paclitaxel plus gemcitabine or FOLFIRINOX has been evaluated in Phase II and III trials (NCT01839487, NCT01959139, NCT02715804). Similarly, to target collagen, the combination of simtuzumab with gemcitabine was tested in a Phase II trial (NCT01472198). Furthermore, the FAK inhibitor GSK2256098, combined with trametinib, was assessed in a Phase II trial (NCT02428270).

To target CAF/PSCs, the inhibition of the Hedgehog pathway using various compounds such as vismodegib and sonidegib has been explored in combination with chemotherapy (NCT01485744, NCT01195415, NCT01064622, andNCT01088815). Additionally, other drugs such as ATRA or paricalcitol, in combination with chemotherapeutics, have been evaluated in Phase I and II trials (NCT03307148, NCT03883919, NCT02754726). Similarly, galunisertib, combined with gemcitabine, has been tested in Phase Ib and II trials (NCT01373164). Among these studies, only one was terminated due to the sponsor decision (NCT02715804).

Although the majority of clinical trials have not yielded promising results, recent advancements have led to the identification of new potential targets and the development of novel combinational therapy strategies. Encouragingly, some approaches have shown sufficient success to progress into clinical trials. A summary of ongoing clinical trials targeting the PDAC stroma is provided in Table 2.

**TABLE 2:** Ongoing clinical trials for targeting the PDAC stroma.

Target	Compound	Combination	Phase of trials	Status	Trial number
FAK inhibitor	Defactinib	Pembrolizumab	Recruiting	II	NCT03727880
		Adaptive Stereotactic Body Radiation Therapy	Recruiting	II	NCT04331041
		Gemcitabine, Nab‐Paclitaxel	Recruiting	I, II	NCT05669482
	AMP945	—	Recruiting	I, II	NCT05355298
IL‐6 antagonist	Tocilizumab	Gemcitabine, Nab‐Paclitaxel, Atezolizumab	Active, Not Recruiting	I, II	NCT03193190
Vit D receptor agonist	Paricalcitol	Gemcitabine + Nab‐Paclitaxel	Active, Not Recruiting	I, II	NCT03520790
		Nab‐Paclitaxel, Cisplatin, Gemcitabine	Active, Not Recruiting	II	NCT04054362
		Gemcitabine, Hydroxychloroquine, Nab‐paclitaxel	Active, Not Recruiting	II	NCT04524702
		Nab‐Paclitaxel, Gemcitabine, Cisplatin	Active, Not Recruiting	II	NCT03415854
PSCs	ATRA	Gemcitabine + nab‐paclitaxel	Recruiting	II	NCT04241276
		Nivolumab	Active, Not Recruiting	Early phase I	NCT05482451
Hedgehog Signaling	Vismodegib	Erlotinib Hydrochloride, Gemcitabine Hydrochloride	Active, Not Recruiting	I	NCT00878163
	NLM‐001	Gemcitabine, Nab‐paclitaxel, Zalifrelimab	Active, Not Recruiting	I, II	NCT04827953
	NV‐101 (taladegib)	—	Active, Not Recruiting	II	NCT05199584
Hyaluronan degradation	PEGPH20	Atezolizumab	Active, Not Recruiting	I, II	NCT03193190
		Gemcitabine, Nab‐paclitaxel	Active, Not Recruiting	N/A	NCT02921022

Ongoing trials include:

- FAK inhibition: Defactinib, in combination with chemotherapy, immunotherapy, or radiation therapy, is being tested in Phase I and II trials (NCT03727880, NCT04331041, NCT05669482). Another FAK inhibitor, AMP945, is under evaluation in Phase I and II trials (NCT05355298).
- IL‐6 inhibition: Tocilizumab, an IL‐6 inhibitor, is being tested in combination with gemcitabine, paclitaxel, and atezolizumab (NCT03193190).
- Vitamin D receptor modulation: Paricalcitol, combined with chemotherapy, is being evaluated in multiple Phase I and II trials (NCT03520790, NCT04054362, NCT04524702, and NCT03415854).
- PSC‐targeting strategies: ATRA, in combination with chemotherapy or immunotherapy, are under investigation in early Phase I and II trials (NCT04241276, NCT05482451).
- Hedgehog pathway inhibition: Vismodegib, NLM‐001, and NV‐101, in combination with chemotherapy and other agents, are being studied in ongoing trials (NCT00878163, NCT04827953, and NCT05199584).
- Hyaluronic acid targeting: PEGPH20, in combination with atezolizumab or gemcitabine plus nab‐paclitaxel, remains under evaluation (NCT03193190, NCT02921022).

These ongoing trials reflect the continued effort to overcome the challenges of targeting the PDAC stroma, aiming to enhance therapeutic efficacy and improve patient outcomes.

## 6 Conclusions and Future Perspectives

PDAC remains one of the most lethal cancers, largely due to the complex and hostile TME, which promotes tumor progression, metastasis, angiogenesis, and therapeutic resistance. PSCs, the most abundant stromal cells in the PDAC TME, play pivotal roles in shaping the desmoplastic stroma by synthesizing and producing ECM components, such as collagen, fibronectin, hyaluronic acid, and MMPs. Additionally, PSCs interact with various cells in the TME by secreting cytokines and growth factors and thereby fuel cancer aggressiveness.

However, PSCs are known to exhibit a dual role in PDAC progression, with both tumor‐promoting and potentially tumor‐suppressive functions depending on the microenvironmental context. This duality presents a major challenge in developing effective therapeutic strategies targeting PSCs or ECM components. Although various therapeutic approaches have been developed and tested in preclinical or clinical studies, most have failed to achieve success in the clinical trials. This highlights the inherent complexity of targeting stromal components such as PSCs or ECM products. Additionally, the dense, fibrotic ECM produced by PSCs serves as a physical barrier, impairing drug delivery and limiting the efficacy of conventional therapies. This underlines the urgent need for innovative alternative strategies to improve drug delivery without inducing adverse effects.

In conclusion, future research should prioritize the development of targeted therapies that can selectively inhibit the tumor‐promoting activities of PSCs and ECM components while preserving their tumor‐suppressive functions. Additionally, the identification of reliable and specific biomarkers for patient stratification will be essential to personalize treatment strategies, ensuring that patients benefit from stromal‐targeting therapies with minimal risks. Furthermore, the integration of novel therapeutic modalities, such as immunotherapy, nanotechnology, and combinational therapies, with stromal‐targeting agents may offer promising avenues to enhance treatment responses and overcome current limitations. Ultimately, a deeper understanding of the intricate interactions within the PDAC TME is essential for advancing the therapeutic landscape. Addressing these challenges with innovative approaches has the potential to significantly improve patient outcomes and offer new hope for tackling this devastating disease.

## Author Contributions

S.S. and D.K. contributed equally to conceptualization, literature review, and writing of this manuscript. S.S. led the initial draft preparation and visualization, while D.K. contributed to critical revisions and editing. Both authors reviewed and approved the final version of the manuscript.

## Ethics Statement

The authors have nothing to report.

## Consent

The authors have nothing to report.

## Conflicts of Interest

The authors declare no conflicts of interest.

## Data Availability

The authors have nothing to report.
